# Supplementary material for: Methylation Risk Score Modelling in Endometriosis: Evidence for Non-Genetic DNA Methylation Effects in a Case–Control Study
Source: Int J Mol Sci. 2025 Apr 16;26(8):3760. doi: 10.3390/ijms26083760 (PMC12027649; doi:10.3390/ijms26083760)
Supplement: Supplementary file 1 [file ijms-26-03760-s001.zip › Supplementary_Figures.pdf]

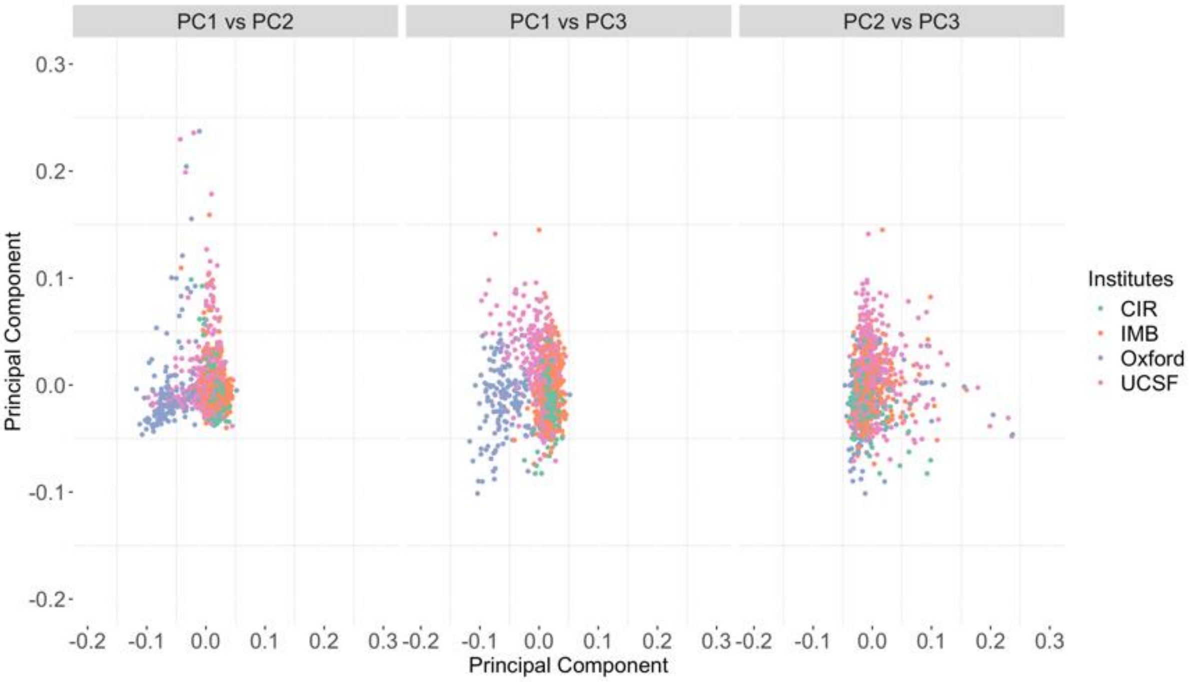

Figure S1. PCA plots showing variation in DNAm profiles of individuals between Institute. Values of the top 3 DNAm PCs were plotted on the x-axis and y-axis. They were plotted against each other in three different combinations, as shown in the title grid at the top of the figure. Points on the scatter plot reflect individual samples coloured according to their Institute, with green, orange, blue and pink representing CIR, IMB, Oxford and UCSF, respectively.

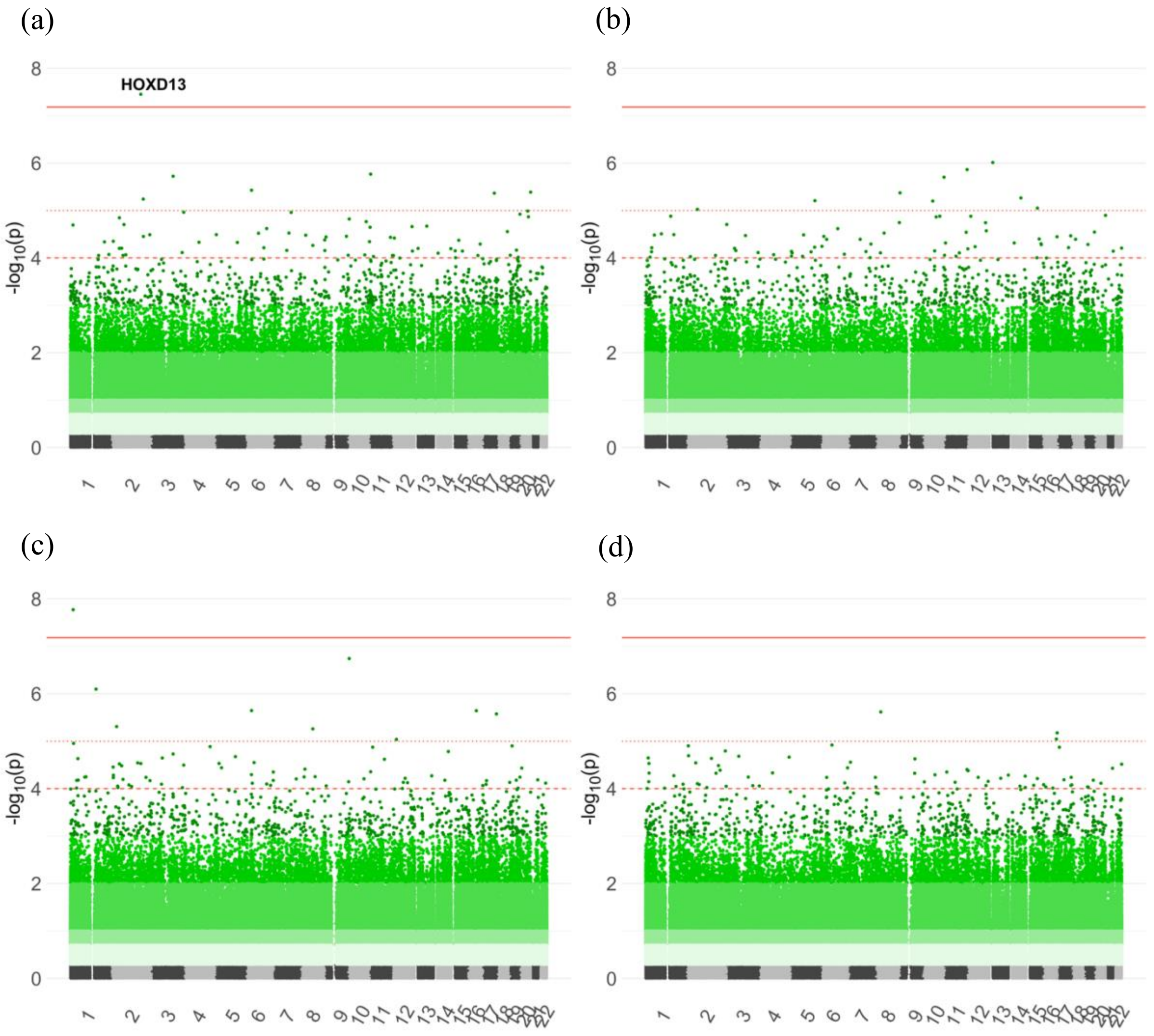

Figure S2. Manhattan plots showing the statistical significance (p-value) of the associations between each DNAm probe (n = 762,651) and endometriosis, generated via MOA on samples from Training Set 1 (a), 2 (b), 3 (c) and 4 (d). Probes that had different p-value thresholds were highlighted in various shades of green with p-values of $p < 0.5$, $p < 0.2$, $p < 0.1$, $p < 0.01$ and $p < 0.001$, represented by the lightest green to the darker green, respectively. $p < 1e\text{-}04$ and $p < 1e\text{-}05$ were highlighted with the darkest green. Red dash and dotted lines represent $p < 1e\text{-}04$ and $p < 1e\text{-}05$, respectively. Genomic inflation factor (λ): Training Set 1 = 1.00; Training Set 2=1.00 (MOA); Training Set 3=1.00 (MOA); Training Set 4 = 1.01 (MOA).

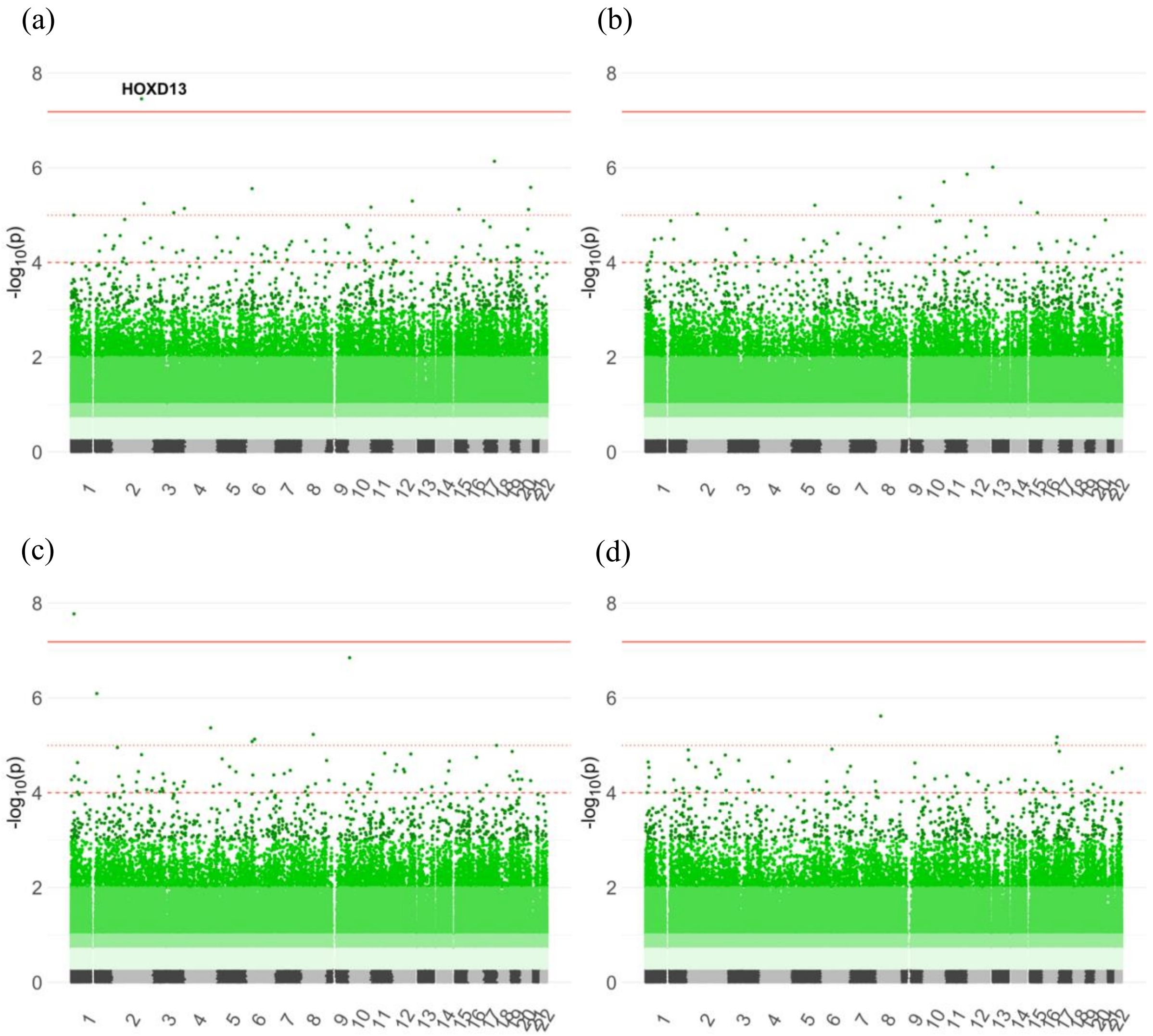

Figure S3. Manhattan plots showing the statistical significance (p-value) of the associations between each DNAm probe (n = 762,651) and endometriosis, generated via MOMENT on samples from Training Set 1 (a), 2 (b), 3 (c) and 4 (d). Probes that had different p-value thresholds were highlighted in different shades of green with p-values of $p < 0.5$, $p < 0.2$, $p < 0.1$, $p < 0.01$ and $p < 0.001$, represented by the lightest green to the darker green, respectively. $p <$ 1e-04 and $p <$ 1e-05 were highlighted with the darkest green. Red dash and dotted lines represent $p <$ 1e-04 and $p <$ 1e-05, respectively. Genomic inflation factor (λ): Training Set 1 = 1.00; Training Set 2= 1.00; Training Set 3= 1.00; Training Set 4 = 1.01.

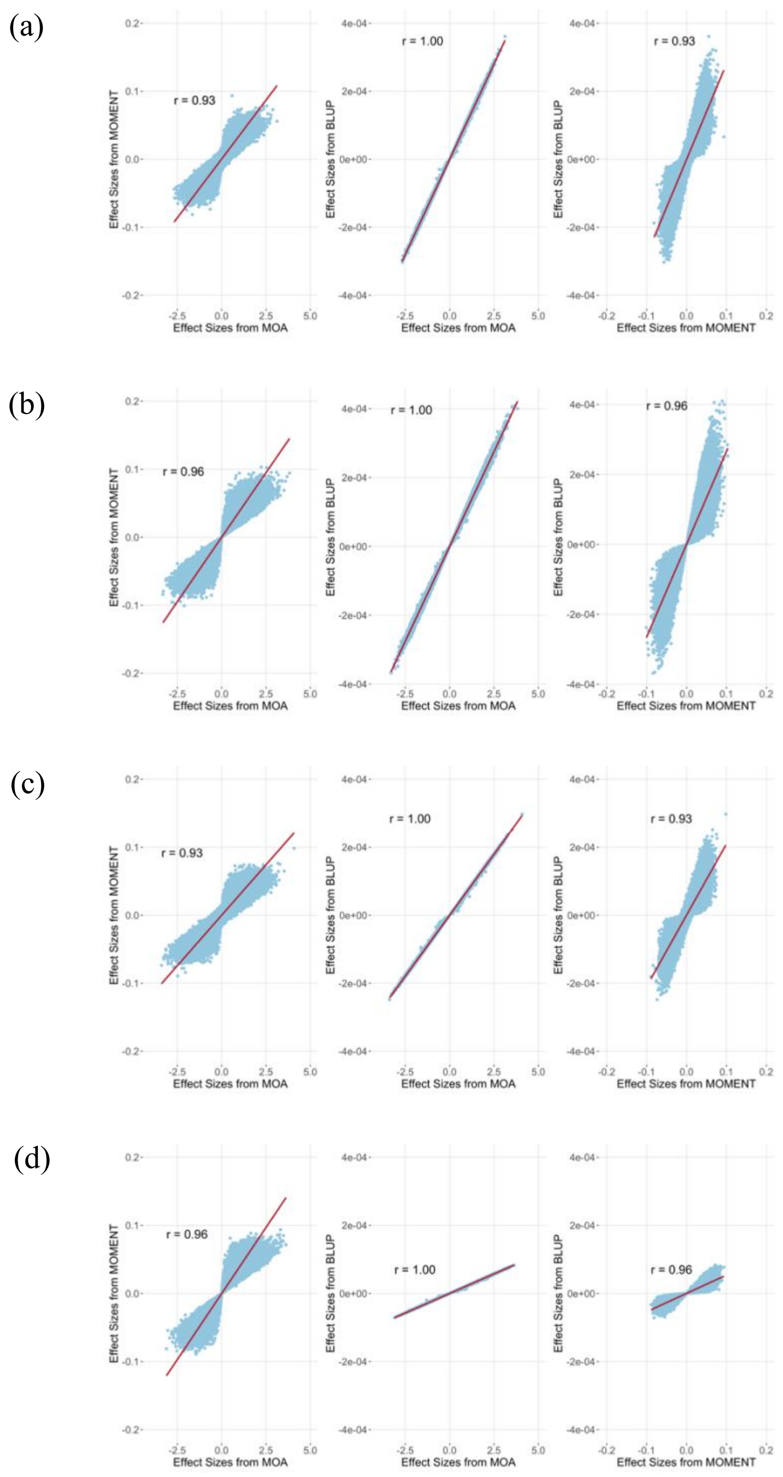

Figure S4. The correlation of effect sizes generated between different methods (MOA, MOMENT, BLUP) on Training Set 1 (a), 2 (b), 3 (c) and 4 (d). Shown from left to right were correlations (r) of effect sizes between MOMENT and MOA, followed by BLUP and MOA, then BLUP and MOMENT. Effect sizes from the corresponding methods were plotted on the X-axis and Y-axis with a solid red line that showed the linear model fit of the relationship.

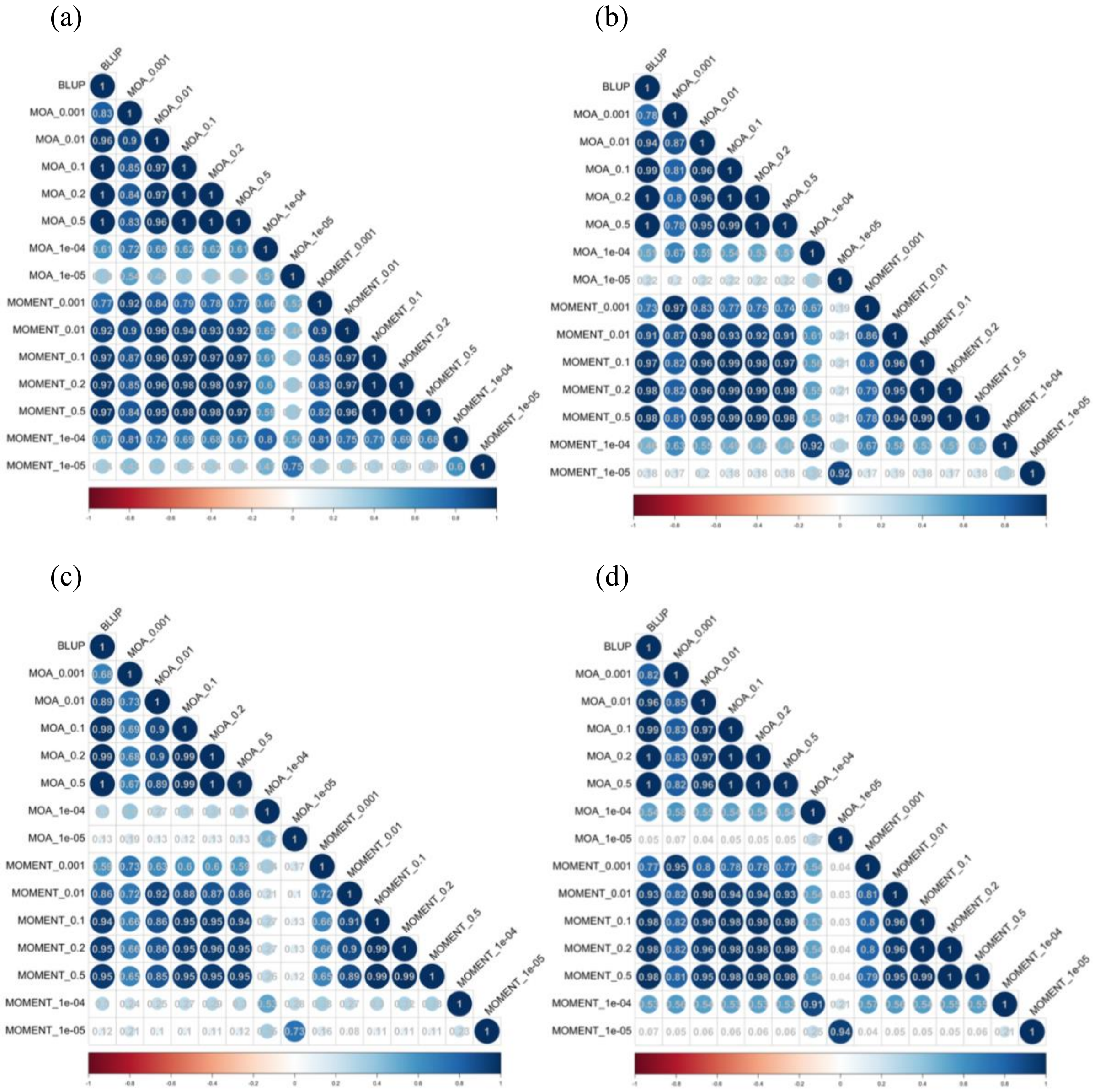

Figure S5. Correlation of MRS across different MRS methods for each test set. Results for CIR, UCSF, Oxford and IMB test sets were illustrated on (a), (b), (c) and (d), respectively. Values on the plot are correlation coefficients. MRS methods labelled on the rows and columns were named according to the three main methods for estimating the effect size of DNAm probes (MOA, MOMENT and BLUP), followed by the p-value thresholds used during probe selection.

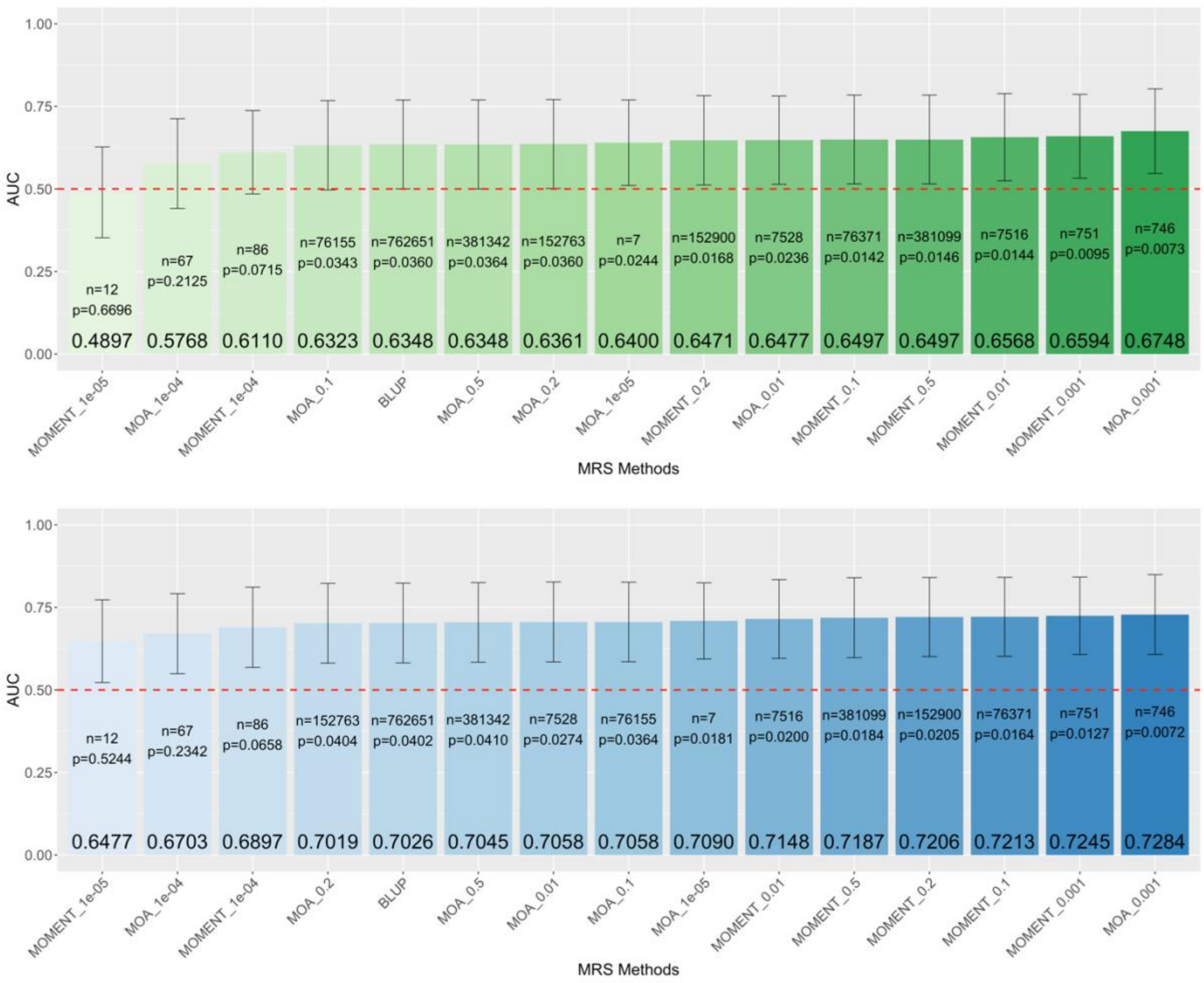

Figure S6. Accuracy across different endometriosis MRS methods with CIR as a test set. Methods used to compute the MRS are MOA, MOMENT, and BLUP. Additionally, DNAm probes for the generation of MRS were selected according to the following p-value thresholds: $p < 0.5$, $p < 0.2$, $p < 0.1$, $p < 0.01$, $p < 0.001$, $p <$ 1e-04 and $p <$ 1e-05 except BLUP, where all DNAm probes were included during computation. MRS methods were labelled on the X-axis according to the three main methods for estimating the effect size of DNAm probes, followed by the p-value thresholds used during probe selection. P-values (p) labelled on each bar were from logistic regression models, Endometriosis status ~ MRS (green bars) and Endometriosis status ~ MRS + PRS (blue bars), and it signifies the statistical significance of the association between endometriosis and MRS. AUCs were plotted on the Y-axis and labelled at the bottom of each bar graph. Number of DNAm probes included denoted by n. The bar graphs were ranked from left to right, from lowest to highest AUC. 95% confidence intervals were indicated by the error bars. Red dashed lines indicated AUC of 0.5.

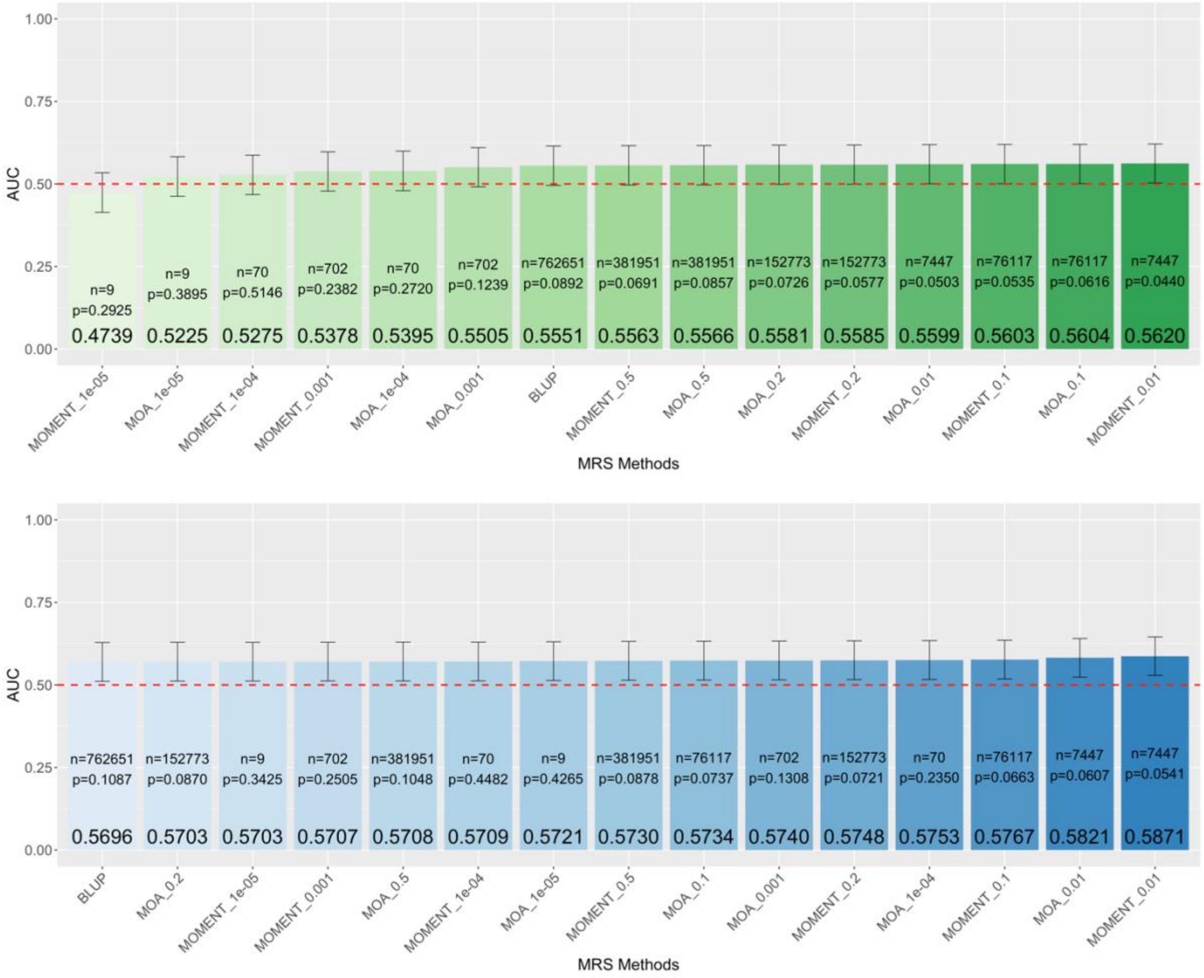

Figure S7. Accuracy across different endometriosis MRS methods with UCSF as a test set. Methods used to compute the MRS are MOA, MOMENT, and BLUP. Additionally, DNAm probes for the generation of MRS were selected according to the following p-value thresholds: $p < 0.5$, $p < 0.2$, $p < 0.1$, $p < 0.01$, $p < 0.001$, $p < 1e\text{-}04$ and $p < 1e\text{-}05$ except BLUP, where all DNAm probes were included during computation. MRS methods were labelled on the X-axis according to the three main methods for estimating the effect size of DNAm probes, followed by the p-value thresholds used during probe selection. P-values (p) labelled on each bar were from logistic regression models, Endometriosis status ~ MRS (green bars) and Endometriosis status ~ MRS + PRS (blue bars), and it signifies the statistical significance of the association between endometriosis and MRS. AUCs were plotted on the Y-axis and labelled at the bottom of each bar graph. Number of DNAm probes included denoted by n. The bar graphs were ranked from left to right, from lowest to highest AUC. 95% confidence intervals were indicated by the error bars. Red dashed lines indicated AUC of 0.5.

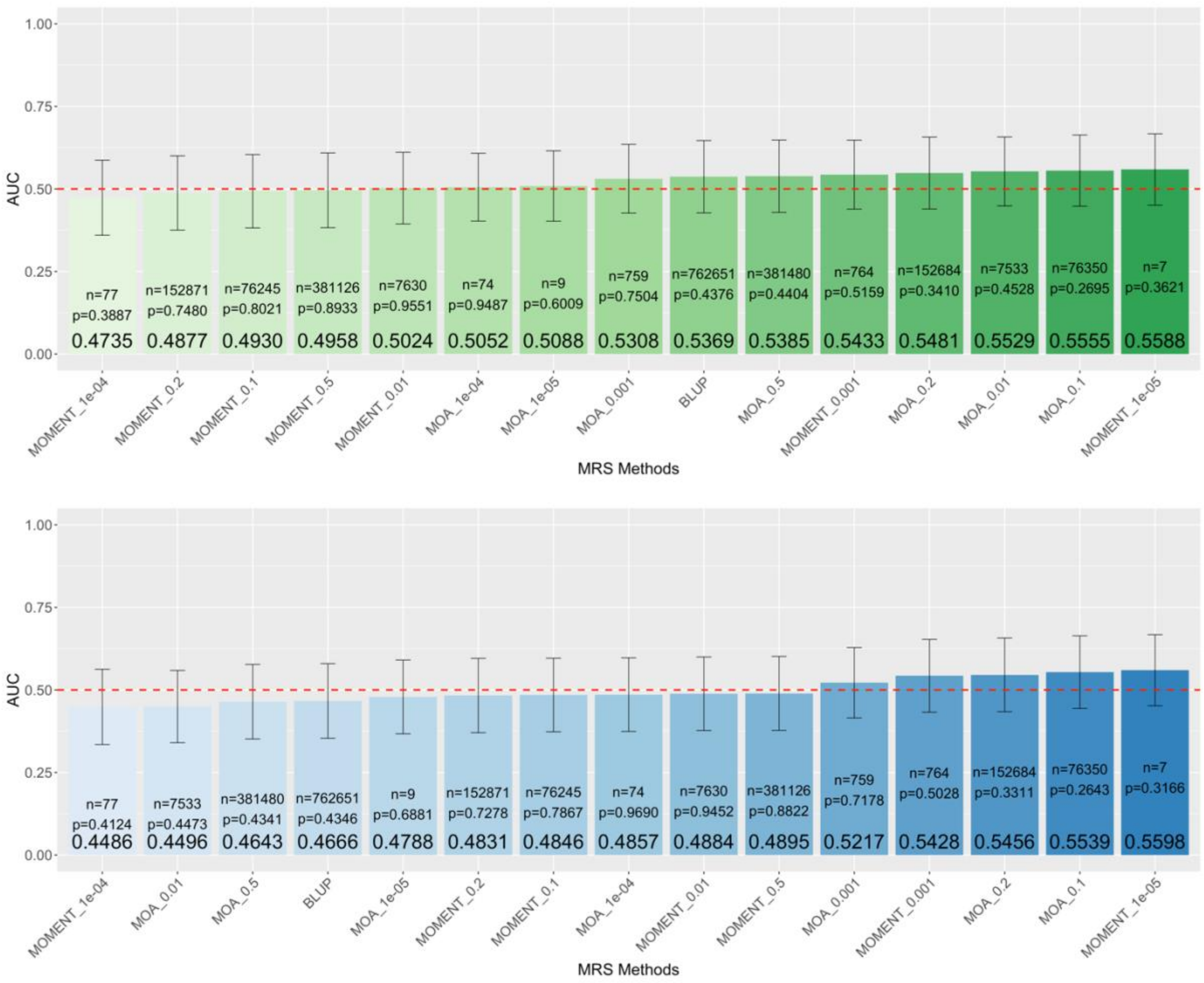

Figure S8. Accuracy across different endometriosis MRS methods with Oxford as a test set. Methods used to compute the MRS are MOA, MOMENT, and BLUP. Additionally, DNAm probes for the generation of MRS were selected according to the following p-value thresholds: $p < 0.5$, $p < 0.2$, $p < 0.1$, $p < 0.01$, $p < 0.001$, $p <$ 1e-04 and $p <$ 1e-05 except BLUP, where all DNAm probes were included during computation. MRS methods were labelled on the X-axis according to the three main methods for estimating the effect size of DNAm probes, followed by the p-value thresholds used during probe selection. P-values (p) labelled on each bar were from logistic regression models, Endometriosis status ~ MRS (green bars) and Endometriosis status ~ MRS + PRS (blue bars), and it signifies the statistical significance of the association between endometriosis and MRS. AUCs were plotted on the Y-axis and labelled at the bottom of each bar graph. Number of DNAm probes included denoted by n. The bar graphs were ranked from left to right, from lowest to highest AUC. 95% confidence intervals were indicated by the error bars. Red dashed lines indicated AUC of 0.5.

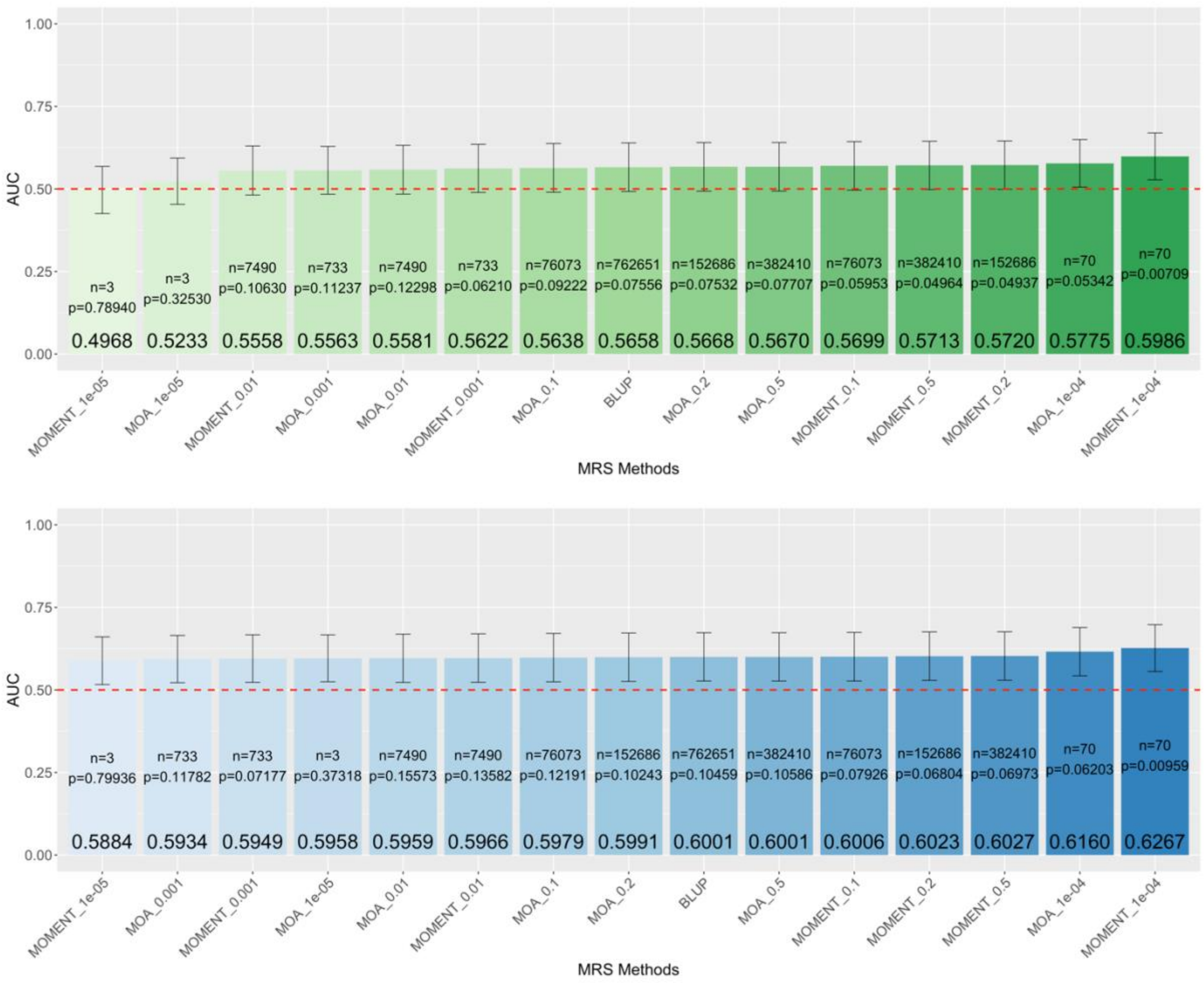

Figure S9. Accuracy across different endometriosis MRS methods with IMB as a test set. Methods used to compute the MRS are MOA, MOMENT, and BLUP. Additionally, DNAm probes for the generation of MRS were selected according to the following p-value thresholds: $p < 0.5$, $p < 0.2$, $p < 0.1$, $p < 0.01$, $p < 0.001$, $p <$ 1e-04 and $p <$ 1e-05 except BLUP, where all DNAm probes were included during computation. MRS methods were labelled on the X-axis according to the three main methods for estimating the effect size of DNAm probes, followed by the p-value thresholds used during probe selection. P-values (p) labelled on each bar were from logistic regression models, Endometriosis status ~ MRS (green bars) and Endometriosis status ~ MRS + PRS (blue bars), and it signifies the statistical significance of the association between endometriosis and MRS. AUCs were plotted on the Y-axis and labelled at the bottom of each bar graph. Number of DNAm probes included denoted by n. The bar graphs were ranked from left to right, from lowest to highest AUC. 95% confidence intervals were indicated by the error bars. Red dashed lines indicated AUC of 0.5.

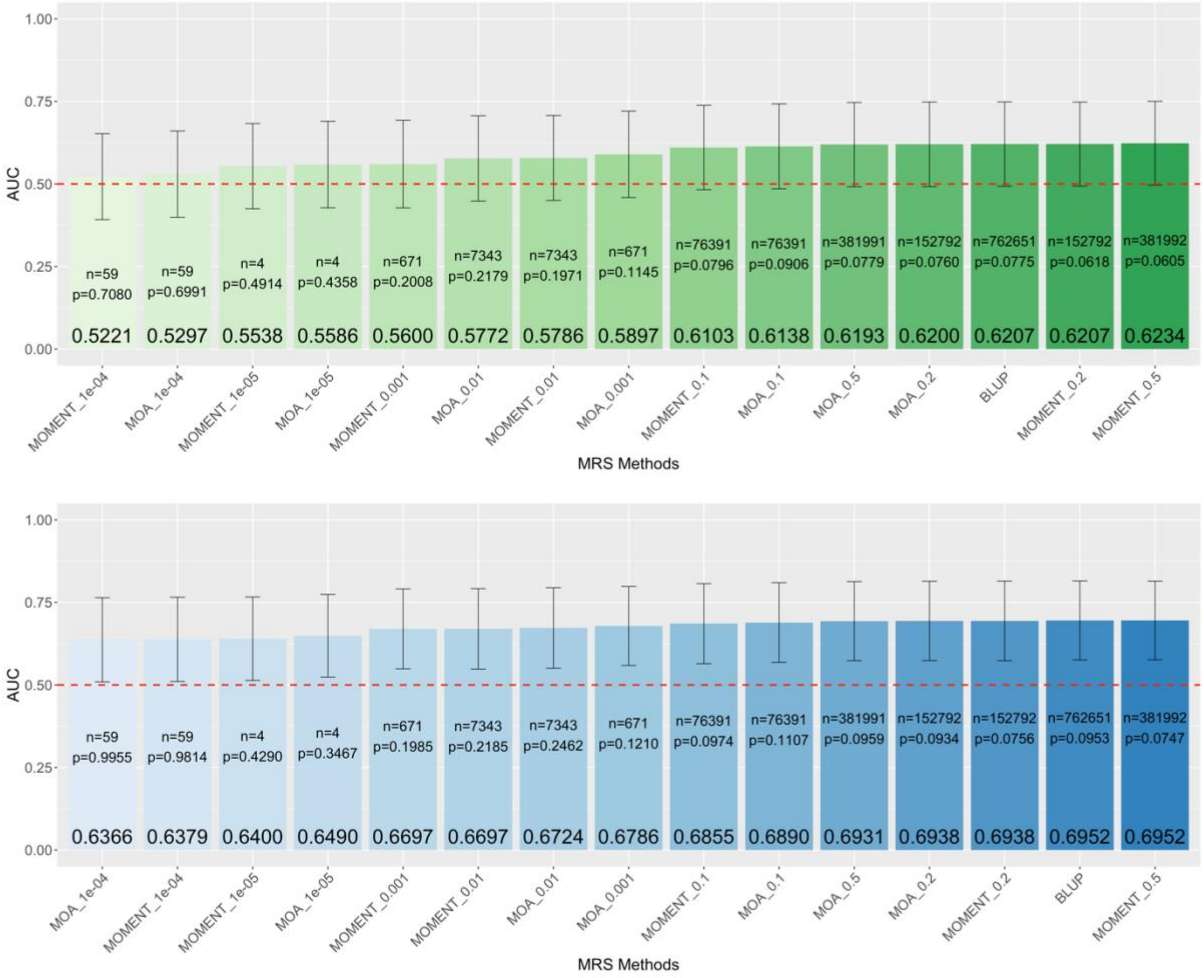

Figure S10. Accuracy across different endometriosis MRS methods with CIR as the test set, using only European genetic ancestry samples. Methods used to compute the MRS are MOA, MOMENT, and BLUP. Additionally, DNAm probes for the generation of MRS were selected according to the following p-value thresholds: $p < 0.5$, $p < 0.2$, $p < 0.1$, $p < 0.01$, $p < 0.001$, $p < 1e\text{-}04$ and $p < 1e\text{-}05$ except BLUP, where all DNAm probes were included during computation. MRS methods were labelled on the X-axis according to the three main methods for estimating the effect size of DNAm probes, followed by the p-value thresholds used during probe selection. P-values (p) labelled on each bar were from logistic regression models, Endometriosis status ~ MRS (green bars) and Endometriosis status ~ MRS + PRS (blue bars), and it signifies the statistical significance of the association between endometriosis and MRS. AUCs were plotted on the Y-axis and labelled at the bottom of each bar graph. Number of DNAm probes included denoted by n. The bar graphs were ranked from left to right, from lowest to highest AUC. 95% confidence intervals were indicated by the error bars. Red dashed lines indicated AUC of 0.5.

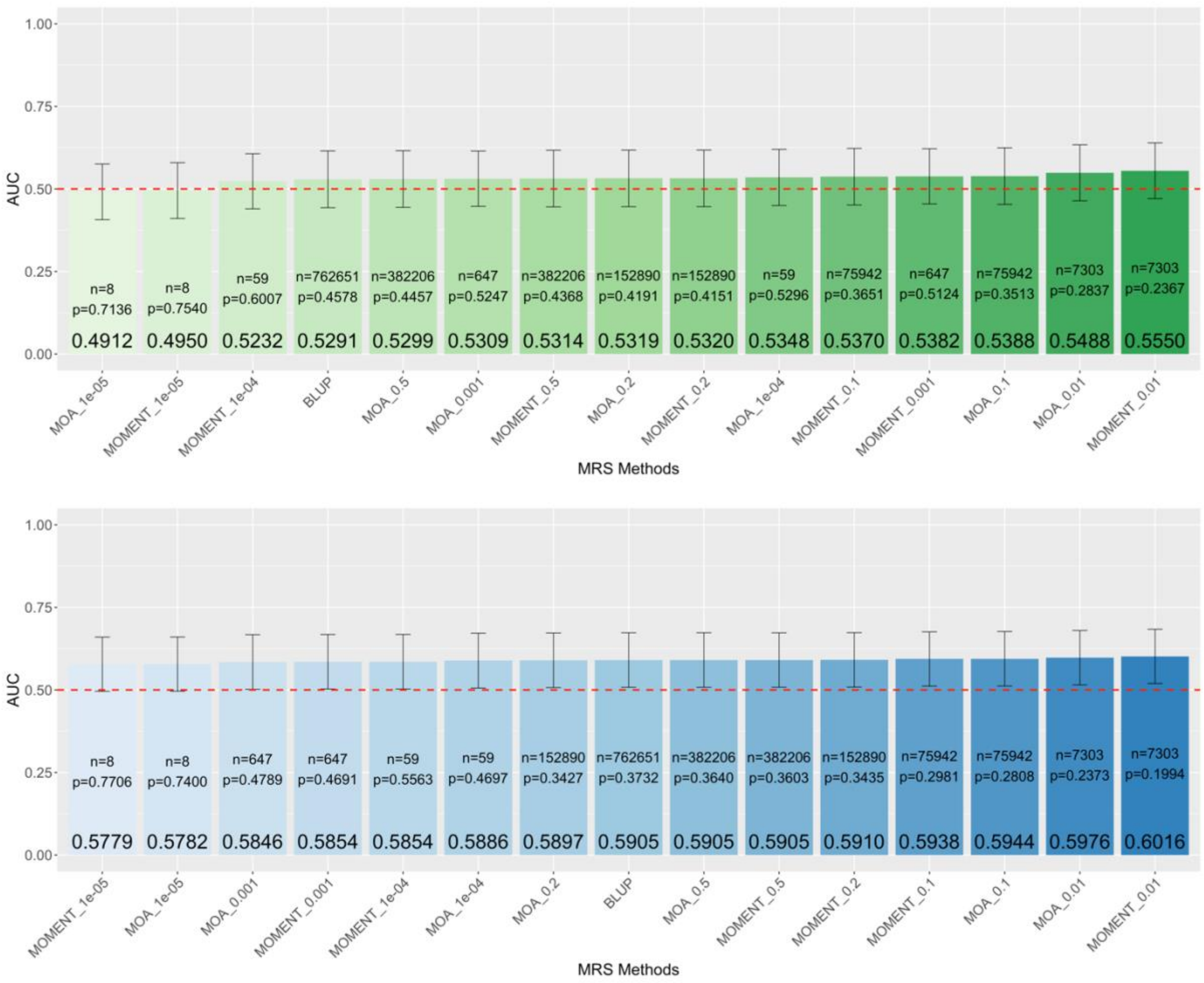

Figure S11. Accuracy across different endometriosis MRS methods with UCSF as the test set, using only European genetic ancestry samples. Methods used to compute the MRS are MOA, MOMENT, and BLUP. Additionally, DNAm probes for the generation of MRS were selected according to the following p-value thresholds: $p < 0.5$, $p < 0.2$, $p < 0.1$, $p < 0.01$, $p < 0.001$, $p < 1e\text{-}04$ and $p < 1e\text{-}05$ except BLUP, where all DNAm probes were included during computation. MRS methods were labelled on the X-axis according to the three main methods for estimating the effect size of DNAm probes, followed by the p-value thresholds used during probe selection. P-values (p) labelled on each bar were from logistic regression models, Endometriosis status ~ MRS (green bars) and Endometriosis status ~ MRS + PRS (blue bars), and it signifies the statistical significance of the association between endometriosis and MRS. AUCs were plotted on the Y-axis and labelled at the bottom of each bar graph. Number of DNAm probes included denoted by n. The bar graphs were ranked from left to right, from lowest to highest AUC. 95% confidence intervals were indicated by the error bars. Red dashed lines indicated AUC of 0.5.

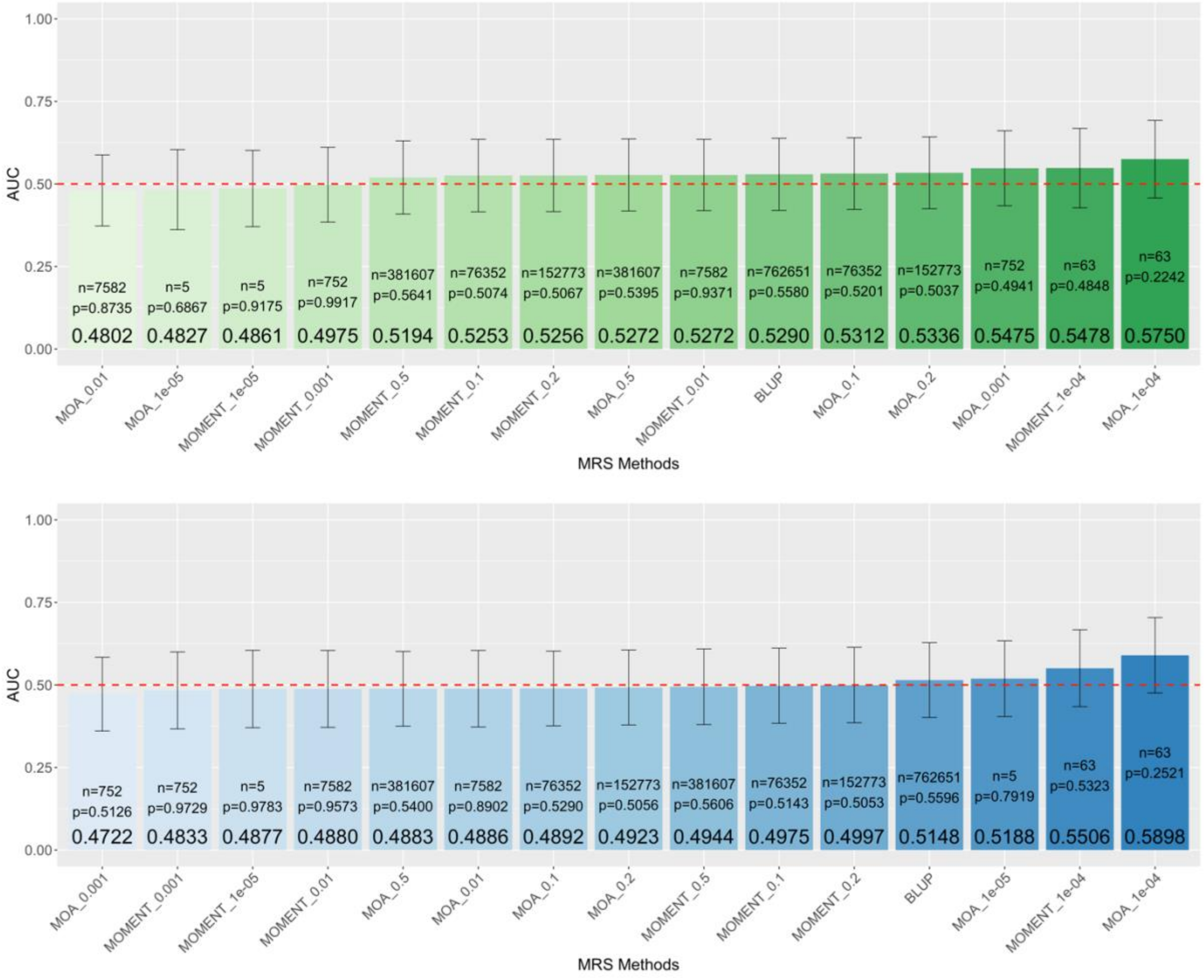

Figure S12. Accuracy across different endometriosis MRS methods with Oxford as the test set, using only European genetic ancestry samples. Methods used to compute the MRS are MOA, MOMENT, and BLUP. Additionally, DNAm probes for the generation of MRS were selected according to the following p-value thresholds: $p < 0.5$, $p < 0.2$, $p < 0.1$, $p < 0.01$, $p < 0.001$, $p <$ 1e-04 and $p <$ 1e-05 except BLUP, where all DNAm probes were included during computation. MRS methods were labelled on the X-axis according to the three main methods for estimating the effect size of DNAm probes, followed by the p-value thresholds used during probe selection. P-values (p) labelled on each bar were from logistic regression models, Endometriosis status ~ MRS (green bars) and Endometriosis status ~ MRS + PRS (blue bars), and it signifies the statistical significance of the association between endometriosis and MRS. AUCs were plotted on the Y-axis and labelled at the bottom of each bar graph. Number of DNAm probes included denoted by n. The bar graphs were ranked from left to right, from lowest to highest AUC. 95% confidence intervals were indicated by the error bars. Red dashed lines indicated AUC of 0.5.

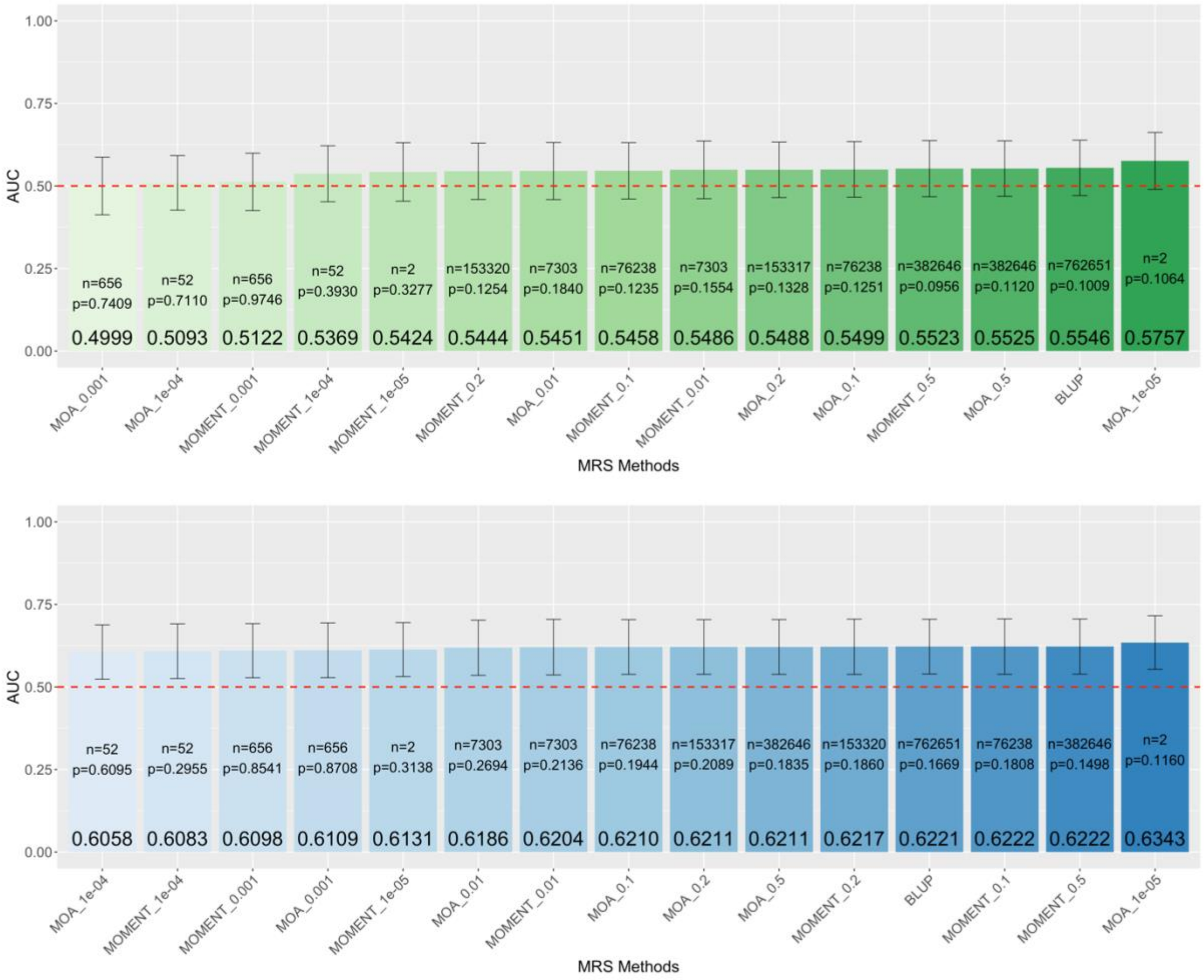

Figure S13. Accuracy across different endometriosis MRS methods with IMB as the test set, using only European genetic ancestry samples. Methods used to compute the MRS are MOA, MOMENT, and BLUP. Additionally, DNAm probes for the generation of MRS were selected according to the following p-value thresholds: $p < 0.5$, $p < 0.2$, $p < 0.1$, $p < 0.01$, $p < 0.001$, $p <$ 1e-04 and $p <$ 1e-05 except BLUP, where all DNAm probes were included during computation. MRS methods were labelled on the X-axis according to the three main methods for estimating the effect size of DNAm probes, followed by the p-value thresholds used during probe selection. P-values (p) labelled on each bar were from logistic regression models, Endometriosis status ~ MRS (green bars) and Endometriosis status ~ MRS + PRS (blue bars), and it signifies the statistical significance of the association between endometriosis and MRS. AUCs were plotted on the Y-axis and labelled at the bottom of each bar graph. Number of DNAm probes included denoted by n. The bar graphs were ranked from left to right, from lowest to highest AUC. 95% confidence intervals were indicated by the error bars. Red dashed lines indicated AUC of 0.5.
